# Using intervention mapping to develop ‘Healthy HR’ aimed at improving sustainable employability of low-educated employees

**DOI:** 10.1186/s12889-021-11278-7

**Published:** 2021-06-29

**Authors:** Emmelie Hazelzet, Inge Houkes, Hans Bosma, Angelique de Rijk

**Affiliations:** Department of Social Medicine, CAPHRI Care and Public Health Research Institute, Faculty of Health, Medicine and Life Sciences, Maastricht University, 6200 MD Maastricht, the Netherlands

**Keywords:** Active involvement, Dialogue, Employers, Intervention mapping, Job control, Low-educated employees, Sustainable employability

## Abstract

**Background:**

The perspectives of low-educated employees are often neglected when designing sustainable employability (SE) interventions. As a result, the interventions offered by the employer do often not align with the needs of low-educated employees. This particular group should therefore be actively involved in the process of developing and implementing SE interventions in their work organizations. The current paper describes the development process of a web-based intervention for HR managers and direct supervisors aimed at improving the SE of low-educated employees. This intervention is specifically designed to involve low-educated employees.

**Methods:**

The first four steps of the Intervention Mapping (IM) approach were used to systematically develop the intervention with the active involvement of stakeholders. Step 1 comprised a needs assessment including a literature review, empirical evidence, scoping search and several focus group interviews with employees and with representatives of employers. Step 2 formulated the intervention objective. During step 3, suitable theoretical methods were selected and translated to practical applications. Step 4 involved the development of a web-based intervention by integrating all information from the preceding steps.

**Results:**

The needs assessment indicated that the employees’ active involvement and employees-employer genuine dialogue should be essential characteristics of an SE intervention for low-educated employees. The online toolkit ‘Healthy HR’ (HHR) was developed, which contains eight steps. Each step consists of one or more tasks helping the employer and employees with developing and implementing SE interventions themselves. One or more dialogue-based tools support each task. The leading principle providing structure within HHR was Adapted Intervention Mapping.

**Conclusion:**

Principles of IM appeared to be useful to develop the intervention HHR systematically. This development process resulted in a practical online toolkit that supports employers in the development and implementation of local SE interventions tailored to the needs of low-educated employees. These employees should be actively involved in the process through a dialogue-based approach. By using IM principles, HHR is expected to increase the effectiveness in bettering the health and well-being of low-educated employees.

**Supplementary Information:**

The online version contains supplementary material available at 10.1186/s12889-021-11278-7.

## Introduction

Given today’s rapid ageing workforce and the major technological changes, employees’ sustainable employability (SE) becomes increasingly important for employers [1–3]. Therefore, employers search for approaches to promote healthy, productive, and valuable work in their employees, now and in the future. SE, a subdomain within the field of occupational health (OH), might be a concept of particular relevance for low-educated employees, as – compared to higher-educated employees – these employees have significantly higher risks of poor health, adverse work conditions, and premature labor market exits [4, 5]. Socioeconomic health inequalities remain large [6, 7]. To improve the SE of low-educated employees, the workplace (organizational level) seems to be a suitable setting to reach this particular group [8]. Although these employees show more health problems and often face poor work conditions, they participate less frequently in workplace health interventions [3, 9]. Additionally, when participating, the effectiveness of these interventions is often limited [10]. An alternative approach is needed as they probably need additional support when it comes to improving their health and SE [3, 4, 11]. Interventions with a too narrow base may thus not fit the reality and needs of this group of employees [12].

Three shortcomings are observed in existing SE interventions for low-educated employees. First, many of them are developed without including these employees’ perspectives [12]. Most often, health-promoting changes at the workplace are decided upon in a top-down way, thereby shutting the door on employee participation and ignoring the employees’ voice [13]; this might be particularly disadvantageous for low-educated employees [12, 14]. Second, despite the urgency, there is a lack of well-developed SE interventions for this particular group [4, 15]. Such interventions ought to be based on theory, empirical evidence, and the experiences of the involved stakeholders [15, 16]. To guarantee a systematic development and the involvement of relevant stakeholders, the Intervention Mapping (IM) approach is recommended [17]. Third, employers largely depend on ready-made health programs of external providers, such as consultants and policymakers [13]. However, given that organizational contexts and realities vary, it is important – in a genuinely participatory approach – that employers have a larger say and introduce the development and implementation of SE interventions themselves [8]. This study aims to address these shortcomings.

This paper describes the underlying development process of a web-based intervention (‘Healthy Human Resources’ (HHR)) for employers aimed at improving the SE of low-educated employees using IM. Job control, active involvement, and dialogue between employees and employer have been selected as the core concepts of interest [18]. The literature indicates that these concepts, which partly overlap, contribute to the optimal implementation and effectiveness of SE interventions for this group [19–22]. Job control (or level of autonomy) refers to an employee’s ability to influence his or her work environment and to participate in decision-making on the job, which is related to positive health outcomes [23]. Job control will be stimulated by giving employees an active voice and involving them in a participative role, in other words, by creating opportunities for a genuine dialogue between employees and employer. Furthermore, to systematical develop HHR, the current study used IM which has been successfully applied in previous, evidence-based workplace interventions [15, 24–26]. To systematically integrate the core concepts of interest to lower educated employees in an IM approach is, to our knowledge, innovative for this specific population. In line with the terminology used in the international social epidemiology literature [27], the term “low-educated” was chosen to indicate the target group of this intervention, as all included employees performed low-skilled jobs and the majority was low-educated. The focus of this paper is to describe the development of a web-based intervention using IM and structured according to the first four IM steps (development of an intervention).

## Materials and methods

The development of the intervention builds on the IM approach. The IM approach was originally meant for the development of tailored, theory- and evidence-based community health programs suited to the needs of a specific population and strongly built on stakeholder involvement [17]. It consists of six consecutive steps: (1) needs assessment, (2) formulating intervention objectives, (3) selecting theoretical methods and practical applications, (4) developing the intervention, (5) planning for program adoption and implementation, and (6) planning for evaluation. The results of each step constitute the input for the following step. The present paper describes the development of HHR, that is, IM steps 1 to 4. IM steps 5 and 6 will be published in future papers.

### Participatory development

HHR was developed within a collaborative environment by researchers (authors of this manuscript), supported by an organizational consultant, and five Dutch work organizations deploying low-educated employees: 1) a governmental institution, 2) a cleaning company, with different worksites, 3) a warehouse, 4) a manufacturing company, and 5) a meat-processing company. These organizations were recruited via the researchers’ established networks; Human Resource (HR) managers in the network were contacted. In addition, HR managers of suitable organizations were approached by email. For each organization, the selection of low-educated employees took place on department level. The researchers asked the HR managers to select departments in which employees performed low-skilled jobs. The vast majority of employees working at these departments had lower educational levels, varying from no education to secondary vocational education. Some of the participating work organizations mainly employ uneducated or low-educated employees, while others employ a more heterogeneous group of employees, including a minority of intermediate and higher educated employees, who still perform low-skilled jobs. The organizational sizes varied from 40 to almost 4000 employees. In four of the five organizations, the employees mainly performed physically demanding work, while the employees in organization 1 performed relatively simple administrative tasks (deskwork). All organizations have a relatively high percentage of sickness absence (> 10%, including long-term absence) among their low-educated employees and all were interested in improving the health and vitality of these employees. Due to a tense Dutch labor market for low-skilled employees, many employers tend to retain their low-skilled employees. Moreover, a considerable dismissal protection under Dutch legislation for employees (different from flex workers and self-employed) still exists, which is more protective than in other social systems [28]. The strategy to take the employee perspective as a starting point, having access to a self-led intervention (without external consultancy that is without extra costs) and free use of the online toolkit HHR, were the main reasons for organizations to commit to this study.

In each organization, several stakeholders were invited by the research team to participate in the development phase: 1) representatives of the target group of low-educated employees, and 2) HR managers, line managers, and supervisors on behalf of the employer (representatives of the employer and eventually HHR end-users). This study has been approved by the Medical Ethical Committee of the academic hospital in Maastricht, The Netherlands (METC 2017–0311). All participants were asked to sign an informed consent form when they start their participation in the study. Throughout the development phase, one member of the research team created a monthly update in the form of a flyer for the participating organizations. The HR manager or supervisor distributed these flyers among their employees. These flyers aimed to keep all involved employees and other relevant stakeholders informed about the development process of HHR.

### IM step 1: needs assessment

The objective of the first IM step was to assess the current situation with regard to SE in general and the needs of the low-educated employees and representatives of employers within the participating organizations. The needs assessment was conducted via a literature review of empirical studies, a review of the theoretical literature and concepts, a scoping search of available online tools within OH, and interviews and focus groups. The purpose of the literature review was to identify effective SE interventions and potentially effective ingredients of SE interventions. In the review of the theoretical literature, the researchers focused on the three core concepts (job control, active involvement, and dialogue). A scoping search of available online tools in different disciplines was carried out via a web search using different search terms. Four interviews and eleven focus groups with employees and five focus groups with representatives of the employer were conducted in the five collaborating organizations.

#### Focus group participants and procedures

To ensure a safe climate for discussing SE (intervention) aspects, separate focus groups (so called ‘expert groups’) for the employees and the employer’s representatives were organized in each organization. The participants were recruited voluntarily or invited by their supervisor. The low-educated employees in the focus groups were a cross-section of the employee population of the participating departments with regard to variables such as gender, age, and work contract. Every participant signed an informed consent. The focus groups were moderated by two researchers. The duration of the interviews and focus groups varied from 1 to 2 h. The following topics were discussed: 1) their current views, problems, and needs with regard to SE; 2) current ways of communication and dialogue within the organization, and 3) needs and preferences about the content of HHR (see Additional file 1 for the focus group guide). Simultaneously, within each organization, short dialogues were performed with representatives of the employer (most often a human resource (HR) manager). These interviews aimed to discuss background information about the organization, such as its vision and structure. Both interviews and focus group meetings were digitally recorded, and notes were taken during the meetings. The data was transcribed via clean verbatim (e.g. no filler words) and paraphrasing. Data was analyzed thematically by creating mind maps of each organization, and all members of the research team eventually concurred on the themes identified.

### IM step 2: formulating intervention objectives

The aim of IM step 2 was to formulate the intervention objectives. The final intervention objective refers to what should be changed to meet the needs of employees and representatives of the employer, that is HR managers and direct supervisors (hereafter both are used interchangeably), as identified in IM step 1. Necessary behavioral actions were identified at the individual and organizational levels. These actions were needed to achieve the desired change and outcomes, as defined in terms of the three core concepts (job control, active involvement, and dialogue).

### IM step 3: intervention design: select theoretical methods and practical applications

The third IM step involved identifying appropriate theoretical methods and translating them into practical applications that could be used within the intervention. A theoretical method refers to behavioral change methods with a strong theoretical basis [17]. Inspired by input derived from IM step 1, suitable theories were selected. Handbooks were consulted on problem-solving, positive psychology, and organizational change management. By translating the theoretical method into a practical behavior, this also led to practical applications [17]. For instance, problem-solving (a theoretical method) was translated into brainstorming sessions (a practical application).

### IM step 4: intervention production: develop intervention components and materials

In IM step 4, the goal was to apply and integrate the results from IM steps 1–3 into HHR. To ensure that the overall intervention objective fitted both the target population and the organizational context, brainstorming sessions were organized with the research team to outline the final scope, sequence, and layout of HHR. A graphic designer created the lay-out and technological features of HHR in accordance with a design document developed by the research team. The content of HHR was initiated and discussed with all research members, including an organizational consultant. The content was adjusted via an iterative process. A final task in this step was to perform a usability test of HHR on three aspects: its look and feel (the attractiveness and layout of HHR), navigation system, and content. Several stakeholders (*N* = 5) of the participating organizations, an independent researcher, and an independent HR manager tested HHR. The usability test was based on ‘think-aloud interviews’ in which participants tested HHR by thinking out loud while they performed an action [29] and/or filling in a checklist focusing on the aforementioned usability aspects.

In time of the development of HHR and its content, the COVID-19 pandemic occurred and impacted practically all aspects of societies worldwide, including work organizations and employees [30]. Also within Dutch work organizations, the COVID-19 pandemic had large consequences for the processes and operational management, including OH and HRM. Several participating organizations in our study even went into a complete lockdown. Therefore, the researchers assessed whether the content within HHR might be adapted due to the COVID-19 pandemic.

## Results

### IM step 1: needs assessment

Table 1 summarizes the main results of the needs assessment per procedure.

**Table 1:**
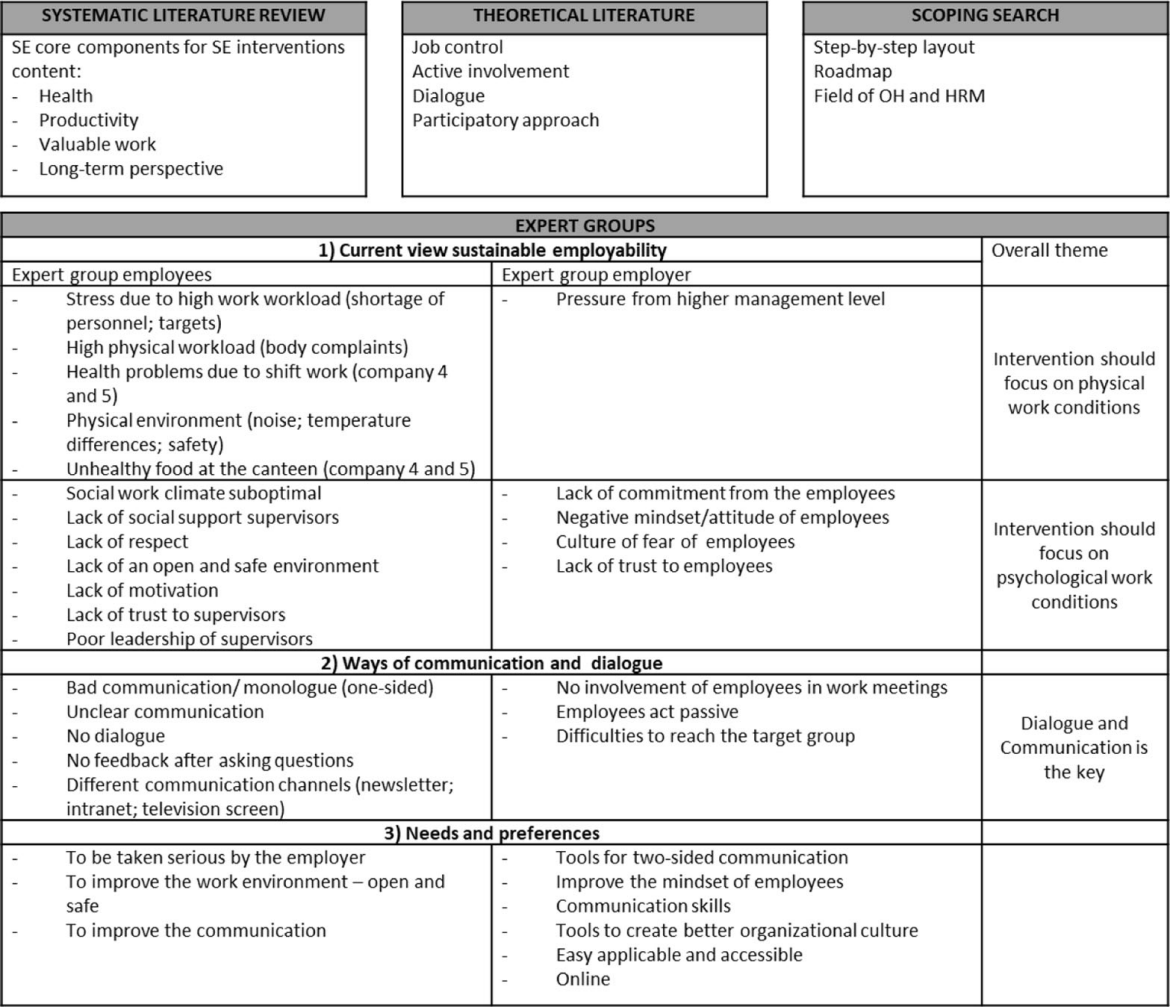
Summary needs assessment

#### Systematic literature review

The systematic literature review about effective employer-initiated SE interventions and potentially effective ingredients of SE interventions has already been published [16]. The review included all interventions framed as SE interventions by the authors themselves, hence the interventions varied widely. In short, based on the results of six intervention studies, it was concluded that a SE intervention should include four SE core components: “health”, “productivity”, “valuable work”, and “long-term perspective”. Considering the content of the interventions evaluated, none addressed all four SE core components. The SE core components “health” and “valuable work” were addressed in all interventions. The “productivity” and “long-term perspective” components were addressed less often. The quality of the evidence for the effectiveness of the interventions was weak to moderate, probably because of inconsistencies in the operationalization of the outcome measures and the lack of an alignment between the intervention content and the outcome measures. One evaluated, moderate-quality study showed a positive effect, possibly resulting from dialogue-based components within the intervention content [31]. The results of the systematic literature review were used to frame SE interventions more clearly and build further on the dialogue-based component used within the content of HHR.

#### Theoretical literature and concepts

A more in-depth review of the theoretical literature on the core concepts (i.e. job control, active involvement, and dialogue) refined our insights. The concept of job control originates from the job demand-control model [23]. For low-educated employees, job control is especially important as they experience low job control in their work, and it is well-known that poor working conditions such as low control at work is associated with health problems and poor health [4, 23, 32–34]. For them, it is very hard to self-direct and to take more job control. They might never have had the opportunity to acquire the skills, means, resilience, and literacy (including health literacy) that are needed for this [35]. The type of work they perform (mainly physical demanding), hierarchical relationships, and the top-down approach within organizations do not easily facilitate job control. However, organizational interventions that include a participatory approach are described as promising solutions to increase job control [19]. Therefore, employees who participate in such interventions get the opportunity to take better self-direction and eventually to experience genuine job control more often, which may eventually improve health and SE.

The second and third concepts are active involvement and dialogue. Workplace interventions are more successful when employees and direct supervisors are truly involved and participate in the initiation phase (i.e. preparation and readiness for change) and active intervention phase (i.e. problem analysis and solving and development and implementation of interventions) [21, 36]. By using the employee’s knowledge (tailoring), this participatory approach leads to an optimization of the fit between the intervention and the organizational context. It also fosters a sense of ownership among employees and creates a positive, collaborative climate between supervisors and employees [24, 37, 38]. Once low-educated employees have been consulted, heard, and truly involved, starting a dialogue and finding solutions together are crucial to improve the effectiveness of interventions [20, 39, 40]. Their self-esteem and self-efficacy are boosted [41], and as the dialogue stimulates mutual trust, the communication, the employees’ work engagement, and perceived working conditions improve [42, 43].

We expect that a participatory approach that integrates a dialogue dimension and actively involves low-educated employees in the decision-making process will lead to increased job control, resulting in improved health and more generally in the promotion of SE [23, 37, 41, 44].

#### Scoping search on existing online tools

The scoping search on the web identified various online tools that have been developed for efficiently supporting human resource management (HRM) and OH [45, 46]. One common theme in these online tools was visualization using a step-by-step plan or roadmap.

#### Focus groups: employees and employer

Physical working conditions, psychological work conditions, and dialogue and communication were identified as the three main themes that are important for SE; the content of HHR should thus focus on these themes. Table 1 summarizes the most important findings per theme per focus group (expert group). As important for their SE, employees mentioned an optimal social (e.g. respect, trust, support, taken seriously) and physical work environment (e.g. noise, temperature). HR managers and supervisors acknowledged that engaging in dialogue with employees is particularly relevant to improve the employees’ SE. However, they mentioned a lack of tools, resources, and expertise to do so. Several aspects were reported as important barriers for the promotion of SE, such as a passive attitude of employees, a traditional company culture (‘work hard and do not complain’), and a lack of time. Moreover, mutual distrust was observed between employees and supervisors. Often the HR managers were the initiators of SE-related projects within organizations. They preferred to involve the direct supervisors as well due to the daily contact and short line with the employees. Difficulties were mentioned in reaching the group of low-educated employees and in effectively communicating with them. Both employees and supervisors often reported poor communication within the organization. Improved communication and dialogue was desired from both parties, but unfortunately often lacking. Finally, HR managers and supervisors found that a website would be the most efficient way to access HHR, because they thought it is easily applicable in daily business.

From IM step 1, we may conclude that it is important that low-educated employees have a say and are actively involved in the intervention development and implementation in their organization. In practice, this appears to be difficult. Therefore, it is important that a web-based intervention should support HR managers and supervisors gradually to facilitate such a process of active involvement and dialogue.

### IM step 2: formulating intervention objectives

The overarching objective of the HHR intervention was formulated as follows: To improve the SE of low-educated employees by supporting HR managers and direct supervisors to involve their employees in developing and implementing tailored SE interventions, with a dialogue-based approach. Further, we hypothesize that the application of HHR within organizations improves the SE of low-educated employees, particularly through increasing the low-educated employees’ control at work.

To meet the overarching intervention objective (improve SE), behavioral and contextual actions are necessary at both the individual (employees and HR managers; supervisors) and the organizational level. At the individual level, all groups need to express positive behavior to improve SE. They need to share the overarching objective by becoming aware of the advantages of HHR. Behavioral actions on the HHR process level (to develop and implement tailored SE interventions) have to take this into account as well. All groups need to express a positive attitude to participate as an active member and need to be able to invest to create tailored SE interventions. They need to feel confident to participate in a dialogue. Employees need to express confidence in their ability to take more control and obtain the feeling of ownership. HR managers and direct supervisors need to be able to explain, encourage, and facilitate the dialogue-based process. They should be able to tailor it to the most important problems of the low-educated employees and implement tailored solutions in the workplace. They also have to facilitate commitment and active involvement with all involved stakeholders. Therefore, they, particularly direct supervisors, play a pivotal role in the entire process. At the organizational level, the higher management should be committed to invest in the availability of time, budget, and additional resources for HHR (e.g. a room to meet). It has to offer the HR managers and direct supervisors these resources to use HHR to develop their SE interventions. Furthermore, for the bottom-up approach, a different, non-hierarchical mindset at different organizational levels is needed.

### IM step 3: intervention design: select theoretical methods and practical applications

Given the formulated intervention objective of IM step 2, Adapted Intervention Mapping (AIM) was chosen as the overall theoretical method to structure HHR [24, 47]. Avoiding the rigor of IM, which will not be practically feasible to use by employers, AIM offers a structure to develop and implement tailored SE interventions in organizations [24].

#### Theoretical methods and practical applications

Using AIM as the leading theoretical method within HHR, the researchers organized HHR along eight smaller steps that are easy for HR managers and supervisors to recognize within the context of their usual tasks. Each step consists of several tasks which can be completed by means of tools (practical applications). To identify suitable theoretical methods for each step and task, the researchers consulted behavioral and organizational science theories, such as empowerment theory, social cognitive theory, and the diffusion of innovations theory. Table 2 presents suitable theoretical methods and types of tools (practical applications) for each step within HHR. Methods of well-known, fundamental theories within IM for behavior change are selected, as well as of theoretical methods other than related to IM. For example, organizational theories informed our use of participatory problem-solving as a theoretical method for HHR steps 2 to 5. This method helps the direct supervisor and employees to translate the problems identified in the needs assessment into potential solutions, to prioritize, and to make an action plan [17]. The citizen participation ladder of Arnstein [51] and the communication framework by Quirke [52] are consulted to help the HR manager or supervisor to identify the level of employee involvement in each task. Moreover, other theoretical methods were identified (not from IM), for instance shared decision-making. This method is in particular beneficial for lower socioeconomic status groups [22].

**Table 2:** Overview of each steps within HHR: goal, theoretical methods and type of tools

Steps HHR	Goal	Theoretical methods (related theory^a^)	Type of tools (Practical applications)
Step 1 Prepare together	Process preparation of HHR by the HR manager to involve and commit all relevant stakeholders	Participation (ET) Facilitation (SCT) Persuasive communication (SCT)	Communication tips and information (guidelines) Fill-in templates
Step 2 Measuring is knowing	Prepare and conduct a needs assessment	Participation (ET) Participatory problem-solving (OT) Persuasive communication (SCT)	Checklist Communication tips and information (guidance) Fill-in templates Questionnaire
Step 3 Our problems	Brainstorm, discuss, and prioritize the problems identified during needs assessment	Participation (ET) Participatory problem-solving (OT) Persuasive communication (SCT) Shared decision-making [22, 48]^b^ Problem-based learning [49]^b^ Self-developed methods^b^: education and trainers’ material	Working format Checklist Communication tips and information (guidance) Fill-in templates
Step 4 Our solutions	Brainstorm, discuss, and prioritize solutions for the problems discussed in step 3	Participation (ET) Participatory problem-solving (OT) Persuasive communication (SCT) Shared decision-making Goal-setting (GST)	Library Working format Checklist Communication tips and information (guidance) External links to reliable sources Fill-in templates
Step 5 Action plan	Discuss and set up an action plan	Participation (ET) Participatory problem-solving (OT) Persuasive communication (SCT)	Working format Checklist Communication tips and information (guidance) Fill-in templates
Step 6 Let’s start	Implement and continue the action plan	Participation (ET) Disseminate, adopt, and implement (DIT) Reinforcement (SCT) Persuasive communication (SCT)	Working format Checklist Communication tips and information (guidance)
Step 7 Evaluation	Evaluate and maintain the action plan or successful aspects	Participation (ET) Evaluation Persuasive communication (SCT) Feedback (GST)	Working format Checklist Communication tips and information (guidance)
Step 8 Along the way: Obstacles in process	Perform a sound dialogue and good cooperation	RDIC model [50]^b^ Persuasive communication (SCT)	Working format Communication tips and information (guidance)

^a^Related theories of the theoretical methods: *SCT* Social Cognitive Theory, *ET* Empowerment Theories, *OT* Organizational Theories, *DIT* Diffusion of Innovations Theory, *GST* Goal Setting Theory. ^b^No theoretical method within IM

Next, the researchers brainstormed about how to translate the suitable theoretical methods into tools (Table 2). For example, for participatory problem-solving (a theoretical method), the researchers included different working formats (tools) in HHR step 3 (our problems), which support the HHR-user to facilitate a meeting. Input from the focus groups (IM step 1) provided information about other tools as well. An important need was to adapt the Maastricht Instrument for Sustainable Employability (MAISE-NL), which was recently developed and validated [53] to the language of the target group (tool within HHR step 2), which resulted in an adapted questionnaire. Voting cards (tool within HHR step 4) were also an outcome from the focus groups. The researchers decided that the intervention should comprise different tools (Table 2). Seven tool types were chosen, with one type, ‘Library’, not being based on a theoretical method:

1. Measure (questionnaire) to tap needs and evaluate effects among employees.
2. A working format (e.g. brainstorm technique) based on theoretical methods or experiences of the research members, which can be used during meetings.
3. A checklist, including the most important topics of that task, to support the HHR-user during a meeting or to fulfil a task.
4. Communication tips and information (guidance) based on theoretical methods and evidence-based/sound examples.
5. Links to reliable external and scientific sources.
6. Fill-in templates (e.g. poster) to support the HHR-user during a task or to collect information together with their employees.

Library, including a review of existing solutions (evidence-based), which can be used as a source of inspiration. The library consists of: 1) a variety of simple solutions, which are relatively easy to apply and inexpensive and 2) evidence-based interventions in the work setting.

### IM step 4: intervention production: develop the intervention

**Fig. 1:**
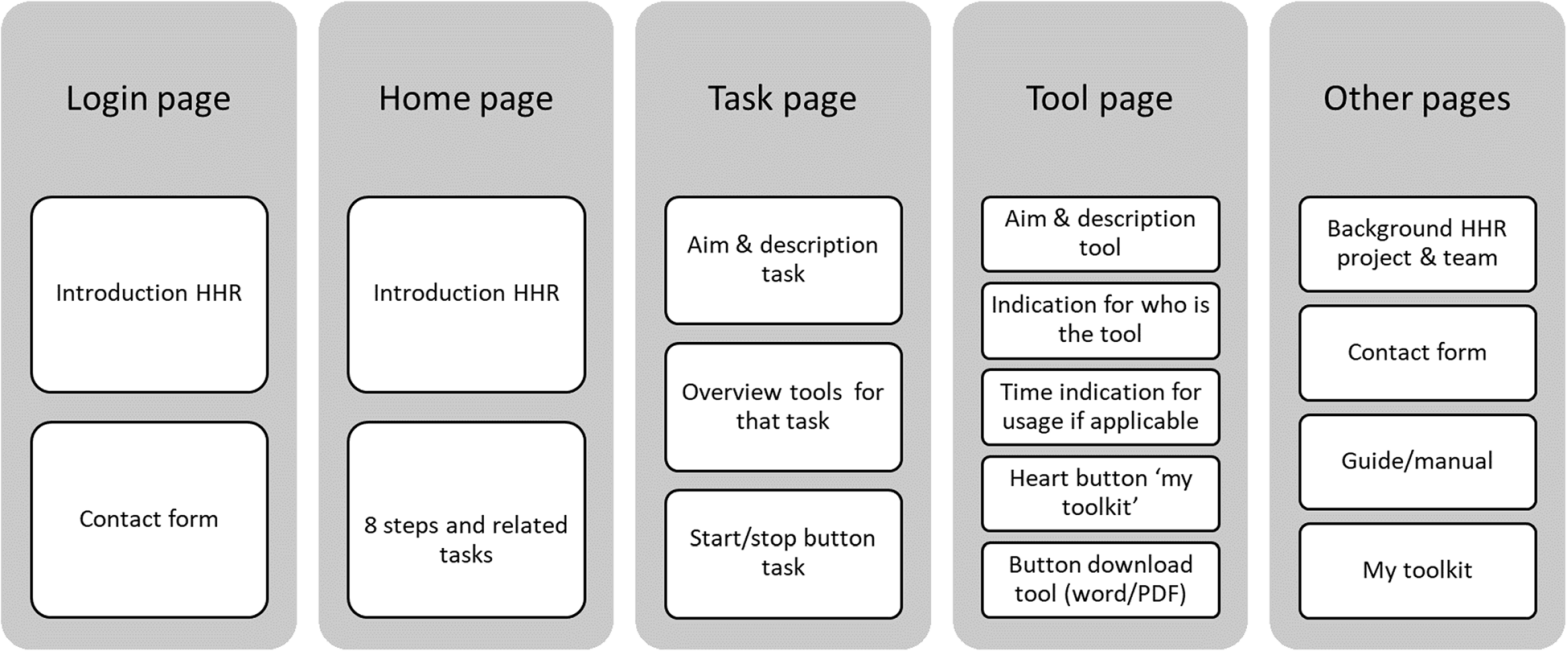
Page structure and content of HHR

The results of the three previous IM steps were operationalized in the HHR intervention along the eight steps and presented via the website, named: ‘Healthy Human Resources’ (HHR) (in Dutch: www.gezondhr.nl). It is assumed that the HR manager initiates HHR. A direct supervisor or an assigned project leader might also apply HHR. A detailed description of the main outline of the steps, tasks, and tools has been published in [18] and added as additional file (See Additional file 2). Figure 1 depicts the page structure and content of HHR. The texts within HHR are easily readable and lack scientific jargon. For all tools, simple and concrete linguistic usage was applied, which is in line with the perceptions and ways of thinking of the low-educated employees*.* A detailed overview is available upon request from the first author (EH).

**Fig. 2:**
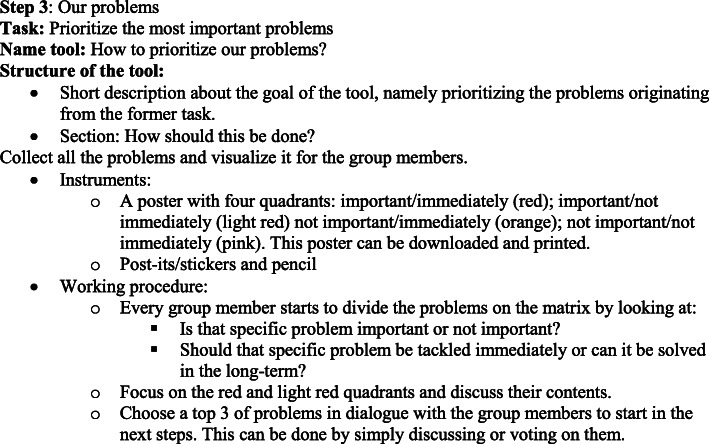
Example tool: How to prioritize our problems?

Figure 2 describes the content of one example tool (tool type 1: working format). Furthermore, HHR-users can select specific tools that best match their context and their employee’s situations to develop a personalized toolkit (‘my toolkit’) for needs assessment and the development and implementation of tailored SE interventions.

#### Testing HHR

After HHR was initially completed, five stakeholders tested its usability. It was perceived as user-friendly, attractive, and a very complete toolkit in general. Based on the usability test, the researchers made minor adjustments to the toolkit, such as simplifying the navigation, adding in a guidance tour about the most common features, and textual changes. Stakeholders indicated that they were not able to judge the content of the tools as they did not yet use them.

#### Adaptations of HHR in the context of the COVID-19 pandemic

The online format of HHR guarantees good access in times of current and future pandemics. New tools for online meetings and information about dealing with the OH aspects of COVID-19 were added. It is our hope that HHR also improves the employees’ say in current and future company-specific, lockdown and lockout measures.

## Discussion

This paper describes the development process of the ‘Healthy Human Resources’ (HHR) intervention. This is an online toolkit to support HR managers and direct supervisors with actively involving their low-educated employees by developing and implementing their own tailored SE interventions in order to improve their SE. HHR was developed using the first four steps of IM and consists of eight steps, each represented by tasks and supportive tools for performing the tasks. The tools can be a questionnaire, working formats, checklists, communication tips and information, external links, fill-in templates, or a library with solutions and interventions.

The development took place in a collaborative environment of researchers, a consultant and employer, and low-educated employees’ representatives from different types of organizations and sectors. The researchers used this participatory approach, which is acknowledged as having the potential to improve the results of organizational interventions [19], right from the beginning. Throughout the development phase, the researchers were constantly in dialogue with the five employers and their low-educated employees. HHR focuses on creating a collaborative environment within an organization in order to develop tailored SE interventions. HHR can be considered as a generic toolkit; it includes a wide range of tools that are specifically aligned to settings with low-educated employees. The HHR-user can select the tools that are applicable in their organization and that best match their group of low-educated employees and their work context.

The traditional IM approach has been proven to be a useful tool to design, implement, and evaluate complex, systematic, theory-based interventions in the field of OH, such as return-to-work programs [45, 54, 55] or workplace health promotion programs [25]. The researchers used the adapted version of IM (AIM) as the leading principle within the intervention itself. AIM is more suitable and practically feasible within a work setting than IM, as former studies showed [24, 47]. By means of AIM, organizations will be able to develop and implement their own tailored SE interventions autonomously.

Through its dialogue-based approach, the HHR intervention is the first systematic online toolkit that – in each and every component of the kit – is aimed at increasing the control of the low-educated employees on the intervention. It offers HR managers and direct supervisors a pragmatic way of working and at the organization level helps them to do a better job at improving the low-educated employees’ SE. The literature and our focus group data revealed that HR managers and direct supervisors often lack the tools and resources to improve SE by themselves [56]. As it aligns to their usual tasks, such as negotiating with higher management, planning and budgeting, we developed HHR for easy adoption by HR. Establishing a true dialogue with the low-educated employees aims to restore the human aspect of HRM, which, we understand, will be new for most of them, especially for the direct supervisors. The toolkit focuses explicitly on facilitating and encouraging active involvement of the low-educated employees during both the choice for SE interventions and the implementation of SE interventions. To optimize this way of working, tools and communication methods are aligned with the way of thinking and needs of low-educated employees. This was based on the needs assessment which indicated that HR managers/supervisors often experience difficulties reaching out to this specific group of employees and communicating effectively with them. Therefore, HR managers and supervisors are encouraged to focus on dialogue through which low-educated employees will get more control over intervention content and implementation. Further, the systematic development process of HHR might inspire researchers in the field of HRM and OH, as it provides important scientific and practical clues for (future) systematic and balanced development of HR interventions. The development of HHR can be seen as evidence-based and evidence-generating. The development phase was based on a combination of empirical evidence and thorough theoretical analyses, and included the perspectives of different stakeholders. Further research on the effectiveness of HHR is needed (IM steps 5 and 6). As one of the objectives of HHR is to develop and implement tailored SE interventions, it also facilitates an evidence-generating aspect. The SE interventions are tailored to the needs of the low-educated employees, which also generates evidence for new out-of-the-box tailored SE interventions. This can be shared as best practices between organizations with low-educated employees and as valuable input for OH research.

Some limitations need to be considered as well. We have chosen to develop and structure HHR as an intervention that can be initiated by an HR manager or supervisor, without involving any external consultancy. However, it is still unknown whether such self-led intervention can be carried out completely without any external consultancy [24]. HHR might be perceived as a ‘disruptive’ intervention as all hierarchical levels are stimulated to transform into active participants and start a dialogue, which will affect the power distribution in the organization – when done as intended – and the organizational culture in the long run. This is especially the case when a direct supervisor is not used to start and continue a true dialogue with the employees. However, if organizations really want to successfully develop and implement SE interventions, safe, open, and supportive workplace cultures are required. Sufficient time, resources, and budget also contribute to the success rate [57]. Although money is saved by not hiring an external consultancy, all employees need to be able to invest a part of their working time to co-create the SE intervention. Additional training on how to deal with HHR might be necessary. Therefore, a process evaluation study is needed to get insight into the barriers and facilitators of the implementation of HHR. Such a process evaluation becomes even more urgent in the current context of the COVID19-pandemic.

To apply HHR implies interdependency between the employees and their supervisors. The idea of HHR is that both parties share their problems and develop and implement solutions together. This ideally leads to a better understanding of each other, but could also enlarge the gap between them. Even when agreements have been made to empower employees and provide them with room for participation, managers might try to bypass these official agreements with informal actions in order to regain power [50]. Supervisors might manipulate HHR to their own advantage, by using their knowledge as a bargaining chip to force employees to take decisions in favor of the employer. This may result in ‘window-dressing’, instead of actual changes in the status quo, and employees are then left with even lower levels of involvement, motivation, and voice. We are aware of the fact that the HHR intervention might create ‘pseudo voice’ [58], that is, managers encourage employees to share their view and pretend to be interested without actually considering their input, because the decisions have already been made. HHR requires integrity and a sincere motivation to improve the SE of low-educated employees. Our research has a humanistic focus, with the aim to highlight employees’ needs and values and giving them a true voice rather than solely aiming at the organization’s existence or profit [59, 60]. Labor unions and local workers’ councils should stay alert to prevent abuse of the toolkit.

## Conclusion

This study described the systematic development of the HHR toolkit. By involving their low-educated employees from the very beginning through an open dialogue, it enables (HR) managers to initiate the development and implementation of tailored SE interventions within their organization. The use of IM resulted in a well-developed intervention, using the principles of IM at two levels: to develop HHR and - using an adapted IM version - as the leading principle within HHR. This study contributes to the need for well-developed and tailored interventions in the field of OH and HRM. The added value of using a theoretical framework and of using IM in combination with a participatory development, we hope, has helped to align science within the field of OH to the daily practice in work organizations deploying low-educated employees. We expect the application of the online HHR toolkit to improve the SE of low-educated employees, as they will profit from regaining control over their work and having a true say about their needs.

## Supplementary Information

**Additional file 1.** Focus group guide.

**Additional file 2.** Table: Steps, tasks and tools HHR.

## Data Availability

The datasets generated and/or analyzed during the current study are not publicly available due to the personal and sensitive information from the involved work organizations and their participants (representatives of employers and employees). The data might be traced back to the work organizations and individual respondents. The data is only available after contact with the corresponding author on reasonable request.

## Abbreviations

AIM: Adapted Intervention Mapping
HHR: Healthy Human Resources
HRM: Human resource management
IM: Intervention Mapping
OH: Occupational health
SE: Sustainable Employability
