# Supplementary material for: Using intervention mapping to develop ‘Healthy HR’ aimed at improving sustainable employability of low-educated employees
Source: BMC Public Health. 2021 Jun 29;21:1259. doi: 10.1186/s12889-021-11278-7 (PMC8240435; doi:10.1186/s12889-021-11278-7)
Supplement: Supplementary file 1 — Additional file 1. Focus group guide. [file 12889_2021_11278_MOESM1_ESM.docx]

**Additional file 1**: Focus group guide*

| **Start** | - Welcome - Explanation of the goal of the focus group - Practical issues: Explanation confidentiality and signing the informed consent form - Room for questions |
| --- | --- |
| **Background information** | - Short introduction round to acquire background information, such as age, gender, function, department, type of work. |
| **Discussion topics/questions**** | 1. Discuss the participants' views, problems, and needs with regard to sustainable employability (healthy work)    1. What does healthy work/sustainable employability mean for you? 2. Ways of communication and dialogue within the organization    1. Can you explain the current way of communication within the organization? And between you, your colleagues and your employer (e.g. supervisor/ HR manager)?    2. Does a dialogue exist? Any problems with regard to dialogue?       1. If problems exist: what are your thoughts about ways to improve the communication/dialogue? What do you need? 3. Needs and preferences about the content of the intervention Healthy HR    1. What kind of tools are already available in the organization?    2. What kind of tools, skills and/or preconditions do you need? Something else? |
| **Closing** | - How did you experience this meeting? - Room for questions and other remaining additions - Thank you for participation. |

*The same focus group guide was used for the employees as well as for the representatives of the employer.

** Depending on the duration, group size and depth of the discussion within the focus groups, topic 1 was discussed in a first focus group session and topic 2 & 3, in a second focus group session.
