# Supplementary material for: Using intervention mapping to develop ‘Healthy HR’ aimed at improving sustainable employability of low-educated employees
Source: BMC Public Health. 2021 Jun 29;21:1259. doi: 10.1186/s12889-021-11278-7 (PMC8240435; doi:10.1186/s12889-021-11278-7)
Supplement: Supplementary file 2 — Additional file 2. Table: Steps, tasks and tools HHR. [file 12889_2021_11278_MOESM2_ESM.docx]

**Additional file 2**

| Steps | Tasks | Tools |
| --- | --- | --- |
| Step 1 Prepare together | Read the vision of HHR | Information about the roles within HHR  Explanation about level of dialogue and involvement at each step |
|  | Compose a project team | Communication tips & fill-in template to create a project group for development |
|  | Develop a project planning | Information and guidelines for project planning  Information and guidelines for project timeline |
|  | Create commitment and involvement at all levels | Fill-in HHR poster template  Fill-in HHR presentation template  HHR Flyer |
| Step 2 Measuring is knowing | Plan, spread, and conduct needs assessment | Communication tips & checklist to conduct a needs assessment  Checklist privacy  “Healthy at work” Questionnaire |
|  | Analyze results of the needs assessment | Manual questionnaire analysis  Fill-in report template for results of needs assessment for management |
| Step 3 Our problems | Communicate the outcomes of needs assessment to employees | Fill-in presentation template for employees – traffic light model (red - take action; orange - prevent further deterioration; green - maintain) |
|  | Brainstorm about relevant problems other than the results of needs assessment | Communication tips & working format for brainstorming – post-its |
|  | Prioritize the most important problems | Communication tips & working format for prioritizing |
|  | Inform all employees about the problem analyses | Communication tips & fill-in poster template for top 3 problems |
| Step 4 Our solutions | Identify and review existing solutions (evidence-based) | Library: two matrices. Matrix 1 with simple solutions. Matrix 2: examples of scientific evidence-based interventions.  Overview of useful websites  Checklist to develop an intervention by the organization |
|  | Brainstorm about possible solutions | Communication tips & working format to conduct ideas about solutions on the work floor  Working format for brainstorming – post-its & brainwriting |
|  | Prioritize the best fitting solutions | Working format – select top 3 solutions per problem – criteria for prioritizing solutions: feasibility, costs, time, effect.  Working format – formulation of SMART solutions & fill-in template |
|  | Vote by employees on the best solutions | Fill-in voting cards template for employees to vote on the best solution |
|  | Communicate about the selected solutions | Communication tips & fill-in poster template for selected solutions |
| Step 5 Action plan | Decision: How to approach the selected solutions? | Preparation tips for management meeting & fill-in sheet for preparation and decision document  Letter template for management  Communication tips & checklist for adaptation solutions |
|  | Develop an action plan | Communication tips & fill-in sheet action plan (W-questions)  Communication tips & fill-in template to create a project group for implementation |
|  | Communicate about the action plan | Communication tips & fill-in action plan poster template |
| Step 6 Let’s start | Implement the action plan | Communication tips & checklist implementation |
|  | Periodic evaluation | Communication tips and approach for evaluation  Working format for evaluation methods |
| Step 7 Evaluation | Conduct a final evaluation | Communication tips & approach for evaluation  Working format for evaluation methods |
|  | Plan for sustaining the successful solutions | Sustainability checklist |
| Step 8 Along the way: Obstacles in the process |  | Tips of do’s and don’ts within a dialogue  Working format to improve collaboration |
